# Quality of life of colorectal cancer survivors participating in a pilot randomized controlled trial of physical activity trackers and daily text messages

**DOI:** 10.1007/s00520-022-06870-5

**Published:** 2022-02-04

**Authors:** Hilary Chan, Katherine Van Loon, Stacey A. Kenfield, June M. Chan, Emily Mitchell, Li Zhang, Alan Paciorek, Galen Joseph, Angela Laffan, Chloe Atreya, Yoshimi Fukuoka, Christine Miaskowski, Jeffrey A. Meyerhardt, Alan P. Venook, Erin L. Van Blarigan

**Affiliations:** 1 Department of Medicine, University of California San Francisco, San Francisco, CA USA; 2 Division of Hematology/Oncology, Department of Medicine, University of California San Francisco, San Francisco, CA USA; 3 Helen Diller Family Comprehensive Cancer Center, University of California San Francisco, San Francisco, CA USA; 4 Department of Urology, University of California San Francisco, UCSF Box 0560, 16th Street, 2nd Floor, San Francisco, CA 94158 USA; 5 Department of Epidemiology and Biostatistics, University of California San Francisco, San Francisco, CA USA; 6 Department of Anthropology, History, and Social Medicine, University of California San Francisco, San Francisco, CA USA; 7 Department of Physiological Nursing, University of California San Francisco, San Francisco, CA USA; 8 Dana-Farber/Partners CancerCare, Boston, MA USA

**Keywords:** Colorectal cancer, Quality of life

## Abstract

**Purpose:**

There are over 1.3 million colorectal cancer (CRC) survivors in the USA, many of whom report lower health-related quality of life (HRQoL) years after treatment. This study aimed to explore the effect of digital health tools on HRQoL in CRC survivors.

**Methods:**

We conducted a two-arm, randomized controlled trial of 42 subjects who had completed treatment for CRC. Participants in the intervention arm received a Fitbit Flex™ and daily text messages for 12 weeks. HRQoL was assessed as a secondary endpoint in both arms at enrollment and 12 weeks using the Medical Outcomes Study Short Form Survey (SF-36) and the Functional Assessment of Cancer Therapy–Colorectal (FACT-C). Survey score changes from enrollment to 12 weeks were compared between the two arms using independent *t* tests, and scores at enrollment and 12 weeks were compared using paired *t* tests.

**Results:**

An increase in the FACT-C functional well-being subscale was observed in individuals in the intervention arm pre- to post-intervention (median difference, 2; interquartile range (IQR), 1, 4; *P* = .02). Although the between-group comparison was not statistically significant, no change in the functional well-being subscale was observed in the control arm (median difference, 0; IQR, 1, 1; *P* = .71). No other measures of HRQoL appeared to differ within arm across time points or between arms.

**Conclusion:**

A 12-week digital physical activity intervention may improve functional well-being among CRC survivors. Larger randomized studies are needed to determine if digital health tools improve functional well-being among CRC survivors and if this improvement can be sustained over time.

**Trial registration:**

NCT02966054; registration date, November 17, 2016

**Supplementary Information:**

The online version contains supplementary material available at 10.1007/s00520-022-06870-5.

## Introduction

Colorectal cancer (CRC) is the fourth most common cancer diagnosed in the USA [1]. As mortality from CRC decreases, the prevalence of survivors continues to rise, with an estimated 1.3 million CRC survivors in the USA in 2014 [1]. With the growing number of CRC survivors, health-related quality of life (HRQoL) becomes an increasingly important patient reported outcome.

CRC survivors often report lower HRQoL compared to the general population [2]. These differences, particularly in the physical functioning domain, are greatest early after treatment [3, 4]. While fairly high overall HRQoL scores have been reported many years after treatment, deficits in physical, emotional, and social functioning may last for more than 3 years after treatment [5, 6]. Furthermore, some studies suggest that these deficits in HRQoL, specifically in younger CRC survivors, can persist for up to 10 years after diagnosis [7].

The effects of exercise on HRQoL have been well-studied in cancer survivors. A meta-analysis of 40 randomized controlled trials (RCTs), involving all types of exercise, suggested that physical activity in breast, colorectal, head and neck, lymphoma, and other cancer survivors resulted in significantly improved global HRQoL at 12 weeks and 6 months [8]. Similarly, in a recent review of 12 observational studies, physical activity was consistently associated with improved survival outcomes and better HRQoL in CRC survivors. [9] Furthermore, a cross-sectional study of 145 CRC survivors reported that sedentary behavior was associated with significantly reduced global quality of life and high disability [10].

These data come from observational studies with self-reported physical activity as well as randomized controlled trials with supervised exercise interventions. Few studies have examined digital health tools that promote physical activity (e.g., physical activity trackers and daily text messages) in relation to quality of life in CRC survivors. We hypothesized that such tools may be a low-cost intervention that improves physical activity and HRQoL in this population. Thus, we included HRQoL as a secondary endpoint in our pilot randomized controlled trial testing the feasibility and acceptability of a digital physical activity intervention in CRC survivors.

Smart Pace was a 1:1 pilot randomized controlled trial of 42 CRC survivors to assess the feasibility of using digital health tools (physical activity trackers and daily text messages). The primary outcome of feasibility was reached, as the intervention was found to be acceptable to CRC survivors [11]. Additionally, while not statistically significant, moderate-to-vigorous physical activity increased by 13 min more per day in the intervention arm compared to that in the control arm. An a priori secondary outcome of this pilot study was to explore the impact of the intervention on HRQoL to inform future studies. In this manuscript, we report on the change in HRQoL among CRC survivors participating in this pilot trial of a digital health intervention.

## Methods

### Study population

This study was a 1:1 pilot randomized controlled trial of 42 CRC survivors to assess the feasibility of digital health tools for promoting physical activity (NCT02966054) [11]. Potential study participants were identified using the University of California, San Francisco (UCSF) Cancer Registry, and through review of provider schedules in the gastrointestinal oncology practice at the UCSF Helen Diller Family Comprehensive Cancer Center. A detailed description of the eligibility criteria has been previously published [11]. Briefly, eligible survivors included those who had colon or rectal adenocarcinoma and were considered disease-free at enrollment. Eligibility criteria also included English reading and writing proficiency, Internet and mobile phone access, and the ability to navigate websites. Exclusion criteria included survivors who were very active at enrollment (defined as self-reported exercise for ≥ 30 min on ≥5 days per week) and those with any medical contraindications to moderate-to-vigorous physical activity (MVPA).

A CONSORT flow diagram can be found in the first publication from this study and is included in Supplemental Fig. 1 [11]. We randomized 42 patients, 21 to the intervention arm and 21 to the control arm. One patient assigned to the intervention arm withdrew from the study prior to receiving the intervention materials after an incidental diagnosis of primary lung cancer, leaving 41 patients who contributed to this analysis of HRQoL. This study was reviewed and approved by the UCSF Institutional Review Board, and all patients provided informed consent prior to enrollment.

### Intervention and control arms

Participants in the intervention arm received a Fitbit Flex™, daily text messages, and print education material. Participants in the control arm received print education material. The intervention content was based on the Theory of Planned Behavior [12]. The content of the text messages and education materials provided participants with informational and motivational messages on various exercise activities, such as brisk walking, jogging, and resistance training. Adherence to the intervention (Fitbit wear, text message responses) was high [11]. During the 12-week study period, participants in the intervention arm wore their Fitbit a median of 74 days (88% of days in the study period; interquartile range (IQR), 23–83 days) [11].

### Quality of life assessments

We measured colorectal cancer-specific quality of life using the Functional Assessment of Cancer Therapy–Colorectal (FACT-C) and general HRQoL using the Medical Outcomes Study Short Form-36 (SF-36) at enrollment and 12 weeks in all participants. Surveys were administered on the web using the UCSF Research Electronic Data Capture (REDCap) system [13]. The FACT-C questionnaire is comprised of 36 questions, 5 subscales, and 3 composite scores. The 5 subscales (physical well-being, social well-being, emotional well-being, functional well-being, and CRC subscale) are a scaled average of 6–7 individual questions [14, 15]. The 3 composite scores (FACT-C total score, FACT-General (FACT-G), and FACT-Trial Outcome Index (FACT-TOI)) are sums of 3, 4, or 5 pertinent subscales. A higher score indicates better HRQoL.

The SF-36 version 1.0 is comprised of 36 questions, 9 domain scores, and 2 component summary scores [16]. The 9 domain scores (physical function, role physical, bodily pain, general health, vitality, social function, role emotional, mental health, and health transition) are comprised of the average of 1 to 10 questions. All 9 domain scores are weighted and combined to calculate both summary scores (physical health component and mental health component). Each summary score is weighted to an average value of 50 and standard deviation of 10 [17]. A higher score indicates better HRQoL. The question “Does your health now limit you in climbing one flight of stairs?” was omitted by mistake from the online REDCap surveys, so the value of that individual item was set to missing for all participants and time points. For the domain physical function (PF) that contained the question about the ability to climb one flight of stairs, we calculated the average score using the available 9 items. All domains and component summary scores were calculable.

### Physical activity tracking

Physical activity was measured at enrollment and 12 weeks using Actigraph GTX3+ accelerometers. All participants were asked to wear the accelerometer on a belt around their waist for 7 consecutive days. Wear time was validated using Troiano 2007 settings in the ActiLife v6.13.3 software [18, 19]. We required at least 10 h of wear time per 24-h period to define a valid day and a minimum of 3 valid days out of the 7 days that the participants were asked to wear the devices. Physical activity was categorized as sedentary (0–99 counts per minute), light (100–2019 counts per minute), moderate (2020–5998 counts per minute), and vigorous (5999 or more counts per minute) using Troiano 2008 cut-points [19]. Our primary measure of physical activity in the pilot study was MVPA, calculated as the sum of time spent performing moderate and vigorous activity.

### Statistical analysis

Survey score changes from enrollment to 12 weeks were compared between the two arms using Wilcoxon signed rank tests, and scores at enrollment and 12 weeks were compared using Wilcoxon rank sum tests. Average effect of intervention on QOL change score was calculated using linear regression as score at 12 weeks minus score at enrollment [20]. SAS® version 9.4 statistical computing software (Cary, North Carolina) was used for analysis, and statistical significance was declared at *p* < 0.05. All analyses were intention-to-treat.

## Results

No differences in age, body mass index (BMI), sex, race, education, employment status, marital status, or stage of diagnosis were found between the intervention and control arms at enrollment (Table 1). By chance, control participants appeared to exercise more at enrollment than the intervention arm participants (mean, 51 min per day compared to 33 min per day, respectively).

**Table 1:** Demographic characteristics, clinical factors, and physical activity at enrollment of 41 colorectal cancer survivors in a pilot randomized controlled trial of a digital health physical activity intervention

Characteristic	Intervention (*n* = 20)	Control (*n* = 21)	*p* value^a^
Age, years, mean ± SD	55.6 (12.3)	54.4 (10.6)	0.755
BMI, kg/m^2^, mean ± SD	29.7 (7.2)	27.1 (4.3)	0.162
Gender, *N*(%)			0.852
Male	8 (40%)	9 (43%)	
Female	12 (60%)	12 (57%)	
Race, *N*(%)			0.680
Asian	2 (10%)	3 (14%)	
Black	1 (5%)	0 (0%)	
Native American, Pacific Islander, or Other	3 (15%)	2 (10%)	
White	14 (70%)	16 (76%)	
College degree, *N*(%)	17 (85%)	21 (100%)	0.065
Works full-time, *N*(%)	14 (70%)	12 (57%)	0.392
Married, *N*(%)	9 (45%)	11 (52%)	0.636
Cancer, *N*(%)			0.890
Colon cancer	11 (55%)	12 (57%)	
Rectal cancer	9 (45%)	9 (43%)	
Years since diagnosis, median [range]	1 [0,8]	1 [0, 4]	0.526
Tumor stage, *N*(%)			0.369
I	4 (20%)	4 (19%)	
II	2 (10%)	6 (29%)	
III	13 (65%)	11 (52%)	
IV	1 (5%)	0	
Moderate-to-vigorous physical activity (minutes/day), mean ± SD^b^	32.9 (17.9)	50.8 (20.7)	0.005
Steps per day, mean ± SD^b^	9008 (3639)	11830 (4052)	0.024

^a^Student’s *t* test or Pearson chi-square test

^b^Average daily moderate-to-vigorous physical activity and steps were measured using 7 days of Actigraph GT3X+ accelerometers

### Functional Assessment of Cancer Therapy–Colorectal

Changes in FACT-C scores within and between treatment arms are summarized in Table 2. No differences were found in any of the FACT-C scores between the intervention and control arm at enrollment. Among participants in the intervention arm, the FACT-C score total score increased by a median difference of 5 (IQR, −5, 9) from enrollment to 12 weeks*.* In the intervention arm, there was an increase in the functional well-being subscale (median difference, 2; IQR, 1, 4) from enrollment to 12 weeks. In addition, in the intervention arm, there was an increase in the FACT-G score (median difference, 6; IQR, −2, 9) from enrollment to 12 weeks. In the control arm, no difference was observed in the functional well-being subscale between enrollment and 12 weeks (median difference, 0; IQR, −1, 1) nor was there a difference in the FACT-G score between enrollment and 12 weeks (median difference, 0; IQR, −8, 7). No other FACT-C subscales, including the CRC subscale, appeared to differ across time points within arm or between arms.

**Table 2:** Colorectal cancer-related quality of life at enrollment and 12 weeks among 41 colorectal cancer survivors participating in a 12-week pilot randomized controlled trial of a physical activity tracker and daily text messages

	Intervention Arm (*n* = 20)				Control Arm (*n* = 21)					
	Enrollment (median [IQR])	12 weeks (median [IQR])	*p* value^a^	Change enrollment to 12 weeks (median [IQR])	Enrollment (median [IQR])	12 weeks (median [IQR])	*p* value^a^	Change enrollment to 12 weeks (median [IQR])	*p* value^b^	Effect^c^ [95% CI]
FACT-C total score	106 [96, 115]	112 [105, 117]	0.22	5 [−5, 9]	107 [92, 114]	104 [93, 115]	0.75	1 [−8, 9]	0.44	2.4 [−5.2, 10.0]
TOI	66 [60, 70]	69 [63, 74]	0.10	3 [−1, 8]	67 [58, 73]	66 [57, 72]	0.98	−1 [−3, 6]	0.20	2.0 [−1.8, 6.4]
FACT-G	87 [79, 91]	90 [87, 95]	0.15	6 [−2, 9]	87 [71, 93]	84 [72, 93]	0.99	0 [−8, 7]	0.23	3.0 [−3.4, 9.5]
Physical well-being	25 [22, 26]	26 [23, 27]	0.29	1 [−1, 3]	24 [22, 27]	25 [23, 26]	0.84	0 [−2, 1]	0.26	0.8 [−0.9, 2.4]
Social well-being^d^	22 [20, 24]	24 [20, 25]	0.58	0 [0, 2]	20 [16, 25]	20 [16, 25]	0.71	0 [−4, 2]	0.56	0.2 [−2.9, 3.2]
Emotional well-being^e^	21 [15, 22]	20 [19, 22]	0.30	1 [−2, 3]	19 [16, 20]	20 [18, 22]	0.050	1 [0, 3]	0.87	0.5 [−2.0, 3.0]
Functional well-being	20 [19, 23]	23 [20, 25]	0.02	2 [1, 4]	21 [16, 26]	21 [16, 25]	0.95	0 [−1, 1]	0.05	2.2 [−0.3, 4.6]
CRC subscale	20 [18, 23]	21 [18, 23]	0.82	−1 [−2, 2]	21 [18, 23]	20 [18, 25]	0.49	0 [−2, 2]	0.49	0.6 [−2.5, 1.2]

^**a**^Wilcoxon signed rank test comparing enrollment and 12-week scores within arms

^**b**^Wilcoxon sum rank test to compare change scores between intervention and control arms

^c^Average effect of intervention on QOL change score calculated as score at 12 weeks minus score at enrollment

^d^In the control arm, the sample size used to calculate the score for social well-being at 12 weeks and change from enrollment was 19

^e^In the intervention arm, the sample size used to calculate the score for emotional well-being at 12 weeks was 16, and the sample size used to calculate change in emotional well-being was 15

### Short Form-36 Vitality Survey

Changes in SF-36 scores within and between arms are summarized in Table 3. Scores at enrollment did not differ between the intervention arm and the control arm. There were no differences in the physical or mental health component summary scores from enrollment to 12 weeks within or between the intervention or control arms. While the median change in the physical sub-score was 0 for both arms, there was a wide range in changes from 0 to 12 weeks for this measure (controls median difference, 0; IQR, 0, 38; intervention median difference, 0; IQR, 0, 33). In addition, there was a suggestion of an increase in the vitality sub-score, a measure of energy and fatigue, observed in the intervention arm from enrollment to 12 weeks (median difference, 10; IQR, 0, 20). No other SF-36 sub-scores differed across time points within arms or between arms.

**Table 3:** General health-related quality of life at enrollment and 12 weeks among 41 colorectal cancer survivors participating in a 12-week digital health physical activity intervention

	Intervention arm (*n* = 20)^a^				Control arm (*n* = 21)^a^					
	Enrollment (median [IQR])	12 weeks (median [IQR])	*p* value^a^	Change enrollment to 12 weeks (median [IQR])	Enrollment (median [IQR])	12 weeks (median [IQR])	*p* value^a^	Change enrollment to 12 weeks (median [IQR])	*p* value^b^	Effect^c^ [95% CI]
SF-36 physical function	89 [81, 94]	94 [83, 100]	0.30	0 [−6, 6]	89 [83, 100]	86 [75, 97]	0.79	−3 [−11, 8]	0.36	3.2 [−7.8, 14.2]
SF-36 role physical	75 [50, 100]	100 [67, 100]	0.41	0 [0, 33]	75 [25, 100]	100 [88, 100]	0.02	0 [0, 38]	0.25	−15.9 [−39.4, 7.6]
SF-36 bodily pain^d^	90 [68, 100]	90 [78, 100]	0.69	0 [−10, 10]	78 [58, 80]	78 [68, 90]	0.86	0 [−23, 23]	0.87	−2.3 [−14.3, 9.6]
SF-36 general health^e^	65 [55, 78]	70 [55, 80]	0.45	5 [−10, 20]	65 [40, 70]	68 [45, 78]	0.16	3 [−4, 10]	0.94	−0.3 [−10.3, 9.6]
SF-36 vitality^e^	54 [50, 65]	70 [60, 80]	0.10	10 [0, 20]	50 [35, 70]	55 [45, 68]	0.33	5 [−8, 11]	0.30	5.4 [−6.8, 17.5]
SF-36 social functioning^e^	88 [75, 100]	88 [75, 100]	0.33	0 [−13, 13]	75 [75, 100]	88 [63, 100]	0.37	0 [−13, 19]	0.30	−6.5 [−16.9, 3.9]
SF-36 role emotional^e^	100 [67, 100]	100 [100, 100]	0.13	0 [0, 33]	100 [67, 100]	100 [100, 100]	0.13	0 [0, 0]	0.74	3.0 [−15.1, 21.1]
SF-36 mental health	80 [70, 88]	84 [72, 92]	0.42	0 [−4, 4]	80 [68, 84]	76 [70, 88]	0.51	0 [−4, 6]	0.96	0.9 [−6.7, 8.4]
SF-36 health transition (1 yr ago)	75 [50, 75]	100 [50, 100]	0.44	0 [0, 25]	75 [50, 100]	75 [63, 100]	0.35	0 [0, 25]	0.63	1.6 [−15.1, 18.2]
Mental health component summary^d^	52 [40, 56]	53 [47, 57]	0.33	2 [−3, 5]	53 [43, 57]	54 [41, 58]	0.46	1 [−1, 2]	0.61	1.8 [−3.5, 7.1]
Physical component summary^d^	50 [46, 53]	51 [46, 55]	0.78	2 [−3, 3]	46 [42, 50]	48 [44, 53]	0.39	2 [−1, 5]	0.47	−1.6 [−5.9, 2.7]

^**a**^Wilcoxon signed rank test comparing enrollment and 12-week scores within arms

^**b**^Wilcoxon sum rank test to compare change scores between intervention and control arms

^c^Average effect of intervention on QOL change score calculated as score at 12 weeks minus score at enrollment

^**d**^In the intervention arm, the sample size at enrollment was 19, and the sample size at 12 weeks and for the change scores was 17. In the control arm, the sample size at enrollment was 17, the sample size at 12 weeks was 18, and the sample size for change scores was 14

^e^In the intervention arm at 12 weeks and for calculation of change scores, the sample size for was 18

## Discussion

In this pilot RCT, we observed little change in overall HRQoL among CRC survivors participating in 12 weeks of physical activity trackers and daily text messages or usual care. However, we did note small improvements in functional well-being among CRC survivors randomized to the intervention, with no change in this domain among controls.

There may be several reasons for the overall lack of change in HRQoL observed in our pilot study. One explanation is the relatively high rates of physical activity among our study population at enrollment. Almost 90% (36 out of 41) of our survivors met or exceeded the recommended amount of physical activity of 150 min per week for cancer survivors at enrollment based on the accelerometers but were still eligible for participation because they had reported on the screening survey that they engaged in 30 min or more of exercise on fewer than 5 days per week [21]. Thus, participants may already have achieved near maximum benefits in HRQoL from physical activity prior to enrollment, which would minimize any observed effect of the digital health intervention on HRQoL. This finding should be considered in determining eligibility criteria for future larger-scale intervention trials, with consideration for targeting individuals with lower levels of physical activity at baseline.

Relatedly, participants at enrollment in both arms had HRQoL scores similar to the general population. The mean SF-36 physical and mental health component summary scores at enrollment in our participants were within the mean and one standard deviation of normalized scores for the US general population (mean 50 ± 10) [22]. This is consistent with prior studies, which have demonstrated near normalization of global HRQoL scores in CRC survivors 1 year after diagnosis [4]. In addition to enrolling inactive survivors, future studies should also target individuals with low HRQoL at enrollment in order to assess whether these survivors may benefit from a digital health physical activity intervention.

In contrast to the average HRQoL scores observed in our patient population, other studies have reported deficits in specific health domains, such as physical, emotional, and social functioning, years after diagnosis in CRC survivors [2, 5, 7]. While we did not observe a difference in score changes between the intervention and control arm in those domains, we observed a small improvement in functional well-being, a measure of ability to perform normal daily activities, in the intervention arm between enrollment and 12 weeks. Functional well-being differs from physical well-being, which is a measure of a patient’s physical symptoms. It is unclear if the increase in the functional well-being subscale by a median of 2 points (IQR, 1, 4) represents a clinically significant difference in HRQoL. Minimally important differences in FACT-C scores were found to be 5 to 8 points for the FACT-C total score and 2 to 3 points for the CRC subscale; however, other sub-score minimally important differences have not been established [23]. Overall, while we did not observe a change in overall HRQoL, our pilot study findings suggest physical activity trackers and interactive text messaging may improve functional well-being in CRC survivors.

### Limitations

There were limitations to our study. First, as a pilot study evaluating the feasibility of digital health tools, we were limited by sample size and were not powered to find statistical significance in physical activity or quality of life. Second, a question from the SF-36 survey was unintentionally omitted from the online surveys at all time points, which may have introduced measurement error. However, this error was not differential between arms or time points. Third, our study did not collect data on treatment history or the presence of peripheral neuropathy prior to enrollment, which could impact HRQoL. Finally, our study participants were largely white, college-educated patients, which may limit generalizability to the larger population of CRC survivors. Furthermore, we were limited by the high levels of MVPA and average HRQoL among the participants at enrollment. This may reflect selection bias as patients already engaged in exercise may have been more interested in enrolling in this study. In summary, an important lesson learned was that future studies examining the impact of digital health interventions on HRQoL should target inactive CRC survivors with low HRQoL at enrollment.

## Conclusion

In this pilot study, we observed no change in overall HRQoL in CRC survivors randomized to 12 weeks of physical activity trackers and interactive text messaging or usual care. However, we did observe an improvement in functional well-being in the intervention arm. Larger randomized studies are needed to definitively determine if a digital health intervention improves functional well-being among CRC survivors.

## Supplementary Information

ESM 1 (DOC 40 kb)

## Data Availability

Data are available upon request.

## Abbreviations

BMI: body mass index
CRC: colorectal cancer
FACT-C: Functional Assessment of Cancer Therapy–Colorectal
FACT-G: Functional Assessment of Cancer Therapy–General
HRQoL: health-related quality of life
IQR: interquartile range
MVPA: moderate-to-vigorous physical activity
PF: physical function
RCT: randomized controlled trial
SF-36: Medical Outcomes Study Short Form Survey
TOI: trial outcome index
UCSF: University of California, San Francisco
