# Supplementary material for: Quality of life of colorectal cancer survivors participating in a pilot randomized controlled trial of physical activity trackers and daily text messages
Source: Support Care Cancer. 2022 Feb 4;30(5):4557–64. doi: 10.1007/s00520-022-06870-5 (PMC8942969; doi:10.1007/s00520-022-06870-5)
Supplement: Supplementary file 1 — (DOC 40 kb) [file 520_2022_6870_MOESM1_ESM.doc]

**Supplemental Figure 1**. Consort Flow Diagram of a pilot randomized controlled trial of a physical activity intervention among individuals with colon or rectal cancer. Lost to follow-up defined as not completing the 12-week accelerometer assessment.

Assessed for eligibility (n=406)

Not Enrolled (n=364)

- Not meeting eligibility criteria (n=221)
- Not English speaking (n=79)
- Contraindication to exercise (n=47)
- ≥150 min/wk of physical activity (n=41)
- Not UCSF patient (n=20)
- On treatment (n=12)
- Not disease-free (n=9)
- Deceased (n=8)
- No mobile phone or Internet (n=4)
- Incarcerated (n=1)
- Declined (n=92)
- No response to invitation (n=51)

Analyzed (n=20)

Lost to follow-up^1^ (n=0)

Discontinued intervention (n=0)

Allocated to intervention (n=21)

- Received allocated intervention (n=20)
- Did not receive intervention (n=1)

- Diagnosed with lung cancer

Lost to follow-up^1^ (n=2)

- Cancer recurrence (n=1)
- Withdrew (n=1)

Discontinued intervention (n=0)

Allocated to Control (n=21)

- Received allocated intervention (n=21)

Analyzed (n=19)

Randomized (n= 42)
